# Implementing newborn screening for sickle cell disease as part of immunisation programmes in Nigeria: a feasibility study

**DOI:** 10.1016/S2352-3026(20)30143-5

**Published:** 2020-06-23

**Authors:** Obiageli E Nnodu, Alayo Sopekan, Uche Nnebe-Agumadu, Chinatu Ohiaeri, Adeyemi Adeniran, Grace Shedul, Hezekiah A Isa, Olumide Owolabi, Reuben I Chianumba, Yohanna Tanko, Juliet H Iyobosa, Adekunle D Adekile, Olufunmilayo I Olapade, Frédéric B Piel

**Affiliations:** a Centre of Excellence for Sickle Cell Disease Research and Training, University of Abuja, Abuja, Nigeria; b Sickle Cell Disease Desk, Non-Communicable Disease Unit, Federal Ministry of Health, Federal Secretariat, Abuja, Nigeria; c Department of Paediatrics, University of Abuja, Abuja, Nigeria; d Department of Paediatrics, Federal Medical Centre, Keffi, Nigeria; e Department of Health, Gwagwalada Area Council Secretariat, Gwagwalada, Abuja, Nigeria; f Pharmacy Department, University of Abuja Teaching Hospital Gwagwalada, Abuja, Nigeria; g Department of Computer Science, University of Abuja, Abuja, Nigeria; h Department of Paediatrics, Kuwait University, Kuwait; i Centre for Clinical Cancer Genetics and Global Health, University of Chicago, Chicago, IL, USA; j Department of Epidemiology and Biostatistics, School of Public Health, Imperial College London, London, UK

## Abstract

**Background:**

Sickle cell disease is highly prevalent in sub-Saharan Africa, where it accounts for substantial morbidity and mortality. Newborn screening is paramount for early diagnosis and enrolment of affected children into a comprehensive care programme. Up to now, this strategy has been greatly impaired in resource-poor countries, because screening methods are technologically and financially intensive; affordable, reliable, and accurate methods are needed. We aimed to test the feasibility of implementing a sickle cell disease screening programme using innovative point-of-care test devices into existing immunisation programmes in primary health-care settings.

**Methods:**

Building on a routine immunisation programme and using existing facilities and staff, we did a prospective feasibility study at five primary health-care centres within Gwagwalada Area Council, Abuja, Nigeria. We systematically screened for sickle cell disease consecutive newborn babies and infants younger than 9 months who presented to immunisation clinics at these five centres, using an ELISA-based point-of care test (HemoTypeSC). A subgroup of consecutive babies who presented to immunisation clinics at the primary health-care centres, whose mothers gave consent, were tested by the HemoTypeSC point-of-care test alongside a different immunoassay-based point-of-care test (SickleSCAN) and the gold standard test, high-performance liquid chromatography (HPLC).

**Findings:**

Between July 14, 2017, and Sept 3, 2019, 3603 newborn babies and infants who presented for immunisation were screened for sickle cell disease at five primary health-care centres using the ELISA-based point-of-care test. We identified 51 (1%) children with sickle cell anaemia (HbSS), four (<1%) heterozygous for HbS and HbC (HbSC), 740 (21%) with sickle cell trait (HbAS), 34 (1%) heterozygous for HbA and HbC (HbAC), and 2774 (77%) with normal haemoglobin (HbAA). Of the 55 babies and infants with confirmed sickle cell disease, 41 (75%) were enrolled into a programme for free folic acid and penicillin, of whom 36 (88%) completed three visits over 9 months (median follow-up 226 days [IQR 198–357]). The head-to-head comparison between the two point-of-care tests and HPLC showed concordance between the three testing methods in screening 313 newborn babies, with a specificity of 100% with HemoTypeSC, 100% with SickleSCAN, and 100% by HPLC, and a sensitivity of 100% with HemoTypeSC, 100% with SickleSCAN, and 100% by HPLC.

**Interpretation:**

Our pilot study shows that the integration of newborn screening into existing primary health-care immunisation programmes is feasible and can rapidly be implemented with limited resources. Point-of-care tests are reliable and accurate in newborn screening for sickle cell disease. This feasibility study bodes well for the care of patients with sickle cell disease in resource-poor countries.

**Funding:**

Doris Duke Charitable Foundation, Imperial College London Wellcome Trust Centre for Global Health Research, and Richard and Susan Kiphart Family Foundation.

## Introduction

Sickle cell disease is a globally distributed genetic blood disorder of high prevalence in sub-Saharan Africa.^1^ The disease is caused by inheritance of an abnormal β-globin allele carrying the sickle mutation on the *HBB* gene (20A→T; Glu6Val). Nigeria has the highest birth prevalence of sickle cell disease in the world, with an estimated 150 000 annual births of babies with sickle cell anaemia, the most common form of sickle cell disease.^2^ Children with sickle cell disease have repeated episodes of painful crisis, anaemia, and increased susceptibility to infections, with an estimated 50–90% risk of dying before age 5 years.^3^, ^4^ According to WHO estimates, sickle cell disease could account for up to 15% of mortality in children younger than 5 years in Africa,^3^ imposing heavy physiological, mental, and financial burdens on affected individuals and their families. Mortality and morbidity can be substantially reduced by early diagnosis and supportive care.^5^ With access to penicillin prophylaxis, hydroxycarbamide treatment, and chronic transfusion programmes for those at risk of stroke, the outlook for individuals with sickle cell disease has substantially improved in most countries over past decades.^6^, ^7^, ^8^, ^9^ These interventions, along with pneumococcal vaccines,^10^ which are typically available as part of national immunisation programmes, rehydration, and health education, are effective and feasible for children with sickle cell disease, even in resource-limited settings.^11^

**Research in context**

**Evidence before this study**

We searched PubMed on March 15, 2020, with the terms “sickle cell disease” AND “newborn screening” AND “Africa”. Our search returned 47 articles, of which nine were excluded because they focused on non-African countries. All references were published in the past 20 years. 29 (62%) of 47 references retrieved were published after 2016. Of 38 publications included, eight were reviews. Seven references focused on point-of care testing devices. No studies incorporated newborn screening for sickle cell disease into existing immunisation programmes.

**Added value of this study**

We showed the feasibility of implementing a newborn screening programme for sickle cell disease based on easy-to-use point-of-care screening tests in multiple primary health-care settings, alongside an immunisation programme in an area of high prevalence, without substantial investment in equipment or staff.

**Implications of all the available evidence**

Our study provides evidence for the feasibility of implementation of newborn screening to reduce the burden of sickle cell disease in African countries. Point-of-care screening tests provide an affordable, reliable, and easy-to-use method to screen for sickle cell disease, ensuring the earliest diagnosis possible, the highest level of follow-up of participants, access to treatments locally (including penicillin prophylaxis, pneumococcal vaccinations, and hydroxycarbamide) and effective prevention procedures regionally (eg, transcranial doppler for risk of stroke). These outcomes are priorities to reduce the mortality and morbidity of sickle cell disease across sub-Saharan Africa and other countries of high prevalence.

To improve the outcome of patients with sickle cell disease, successful implementation of programmes of screening, education, follow-up, and management is needed.^12^, ^13^, ^14^ Babies with sickle cell disease need to be identified at birth or shortly afterwards by primary screening and confirmatory testing. Prenatal and postnatal education and counselling should be available to parents and relatives. Regular follow-up to monitor disease progression and treatment adherence are essential to prevent severe chronic complications.^11^, ^15^, ^16^

Several pilot programmes for newborn screening have been undertaken across Africa, but up to now these have been relatively small and mostly hospital-based,^17^ with few exceptions.^18^, ^19^ Although some studies have suggested such programmes are cost-effective, particularly when the birth prevalence of sickle cell disease exceeds 0·2–0·3%,^20^ cost-effectiveness largely depends on the affordability and accessibility of the tests, care, and follow-up. In particular, implementation of newborn screening in settings where the haemoglobin S (HbS) variant is rare presents specific challenges.^21^ Furthermore, early detection by newborn screening needs a supportive and functional public health infrastructure to be administered.^21^, ^22^

**Figure 1:**
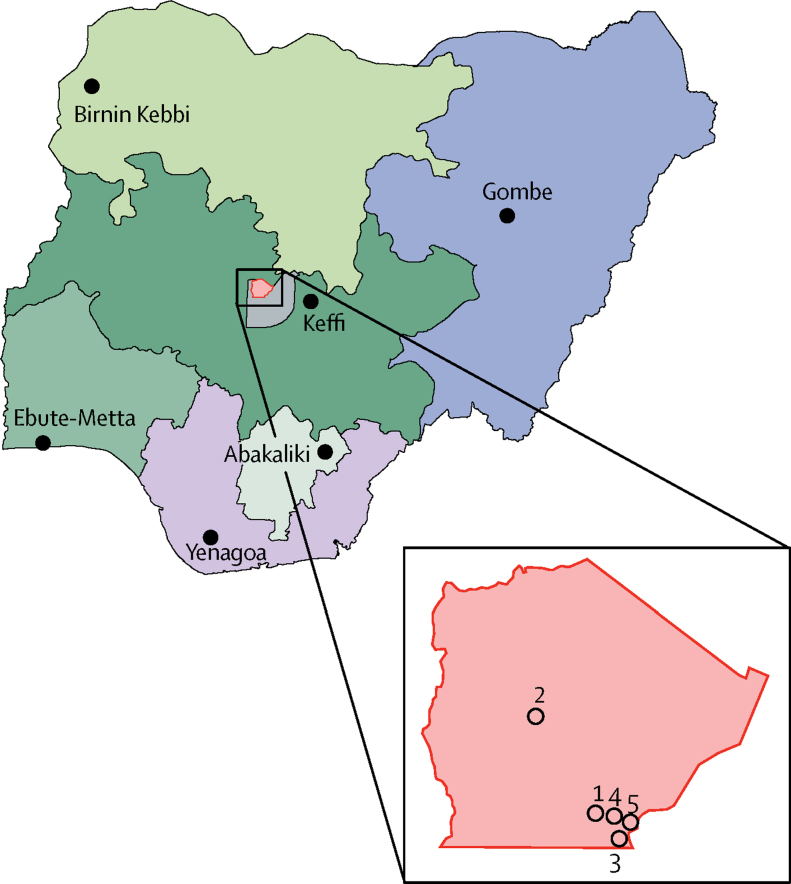
Maps of Nigerian geopolitical zones and locations of study centres Map shows six geopolitical zones in Nigeria and locations (black dots) of Millennium Development Goal sickle cell centres at which high-performance liquid chromatography machines were implemented in 2011–12. The Federal Capital Territory is in the centre of the map and the Gwagwalada Council Area is highlighted in red. Inset map shows locations (open circles) of the five primary health-care centres in the Gwagwalada Council Area that participated in this screening study for sickle cell disease. 1=University of Abuja Teaching Hospital. 2=Dobi Community Clinic. 3=Dagiri Comprehensive Heath Centre. 4=Gwagwalada Township Clinic. 5=Angwan Dodo Public Health Centre.

Traditional diagnostic methods for sickle cell disease include cellulose acetate electrophoresis, isoelectric focusing,^23^, ^24^ tandem mass spectrometry, and high-performance liquid chromatography (HPLC). Each of these methods has important limitations for scaling up to a wide-reaching national programme in low-income settings. The challenges of screening for sickle cell disease with traditional diagnostic methods in Africa are well illustrated by the initiative from the Nigerian Federal Ministry of Health to establish six special Millennium Development Goal (MDG) sickle cell centres across Nigeria between 2011 and 2012. These federal medical centres are located in Birnin Kebbi (Kebbi State in northwest Nigeria), Gombe (Gombe State in northeast Nigeria), Keffi (Nasarawa State in north-central Nigeria), Ebute Metta (Lagos State in southwest Nigeria), Abakaliki (Ebonyi State in southeast Nigeria), and Yenagoa (Bayelsa State in south Nigeria; figure 1). Each centre was equipped with an HPLC machine (Bio-Rad, Hercules, CA, USA). A national protocol for newborn screening for sickle cell disease was developed to guide screening efforts countrywide. By 2017, the number of newborn babies screened was fewer than 2000 across the six MDG sickle cell centres. The main challenges encountered were no specific budgetary allocation, inadequately trained personnel, expired reagents, limited availability of consumables, absence of mechanisms to collect samples from babies on a regular basis, and erratic power supply.

Inexpensive easy-to-use tests, which can differentiate common haemoglobin genotypes in newborn babies and that can be done at remote sites, have been developed.^25^, ^26^ These point-of-care tests are based on different diagnostic principles, such as erythrocyte density, differential mobility of haemoglobin S (HbS) and haemoglobin A (HbA) through filter paper, and antibody-based immunoassay. We aimed to assess the reliability and ease-of-use of two point-of-care screening tests currently available in primary health-care centres and to show the feasibility of a bottom-up approach to implement routine screening, detection, and follow-up of babies with sickle cell disease in primary health-care centres alongside an immunisation programme.

## Methods

### Study design and participants

We did a prospective cohort study at five primary health-care centres within Gwagwalada Area Council, Abuja, Nigeria (figure 1). Steps taken to set up newborn screening and select participating primary health-care centres are described in the appendix (p 2), including surveys of mothers to assess newborn screening acceptability and health-care workers to assess staff resources at health-care centres.

We included all consecutive newborn babies and infants younger than 9 months who presented to immunisation clinics at the five centres. Mothers whose babies had not been screened were counselled and given the opportunity to participate. Basic demographic information and contact details were recorded using questionnaires at enrolment (appendix p 7).

We obtained ethics approval for our study from the University of Abuja Teaching Hospital Health Research Committee (UATH/HREC/469). We obtained written informed parental consent for participation (appendix p 3). To support affected families, registration and first consultation fees were paid for babies of consenting parents at the University of Abuja Teaching Hospital from the Doris Duke Charitable Foundation grant.

### Procedures

All newborn babies and infants younger than 9 months at the primary health-care centres were screened for sickle cell disease using the point-of-care test HemoTypeSC (Silver Lake Research, Azusa, CA, USA). Blood was drawn from each child by heel prick and approximately 1 μL of blood was absorbed into the HemoTypeSC absorbent pad for testing, according to the manufacturer's instructions (appendix pp 4–5). Additional blood spots on filter paper cards (supplied by the Association of Public Health Laboratories; PerkinElmer Health Sciences, Greenville, SC, USA) were collected from each child who tested positive via the HemoTypeSC test. Blood spots were air dried and shipped within a week of collection to the federal medical centre newborn screening laboratory for north-central Nigeria (Keffi, Nasarawa State). Confirmatory testing was done by HPLC testing using the Bio-Rad machine, according to standard protocols.^27^

Results from testing were given to parents by the study nurse who did postscreening counselling. Babies also received pneumococcal and other vaccines as part of the national programme of immunisations at every participating primary health-care centre (appendix p 8). Follow-up visits were scheduled every 3 months after screening. Dedicated pharmacists maintained a register of screen-detected babies with sickle cell disease and supplied folic acid and oral penicillin. Hydroxycarbamide could not be administered as part of this study because funding did not cover this drug. The need to adhere to prescribed drug regimens to reduce complications was highlighted during the first follow-up visit. To administer the drugs, parents or guardians were asked to dissolve penicillin V or folic acid tablets in a 10 mL container before administration. For breastfed babies, we advised that breast milk could be used as a diluent. At each visit, parents or guardians were encouraged to adhere to the drug regimen and not to share drugs with other family members and friends.

Supervising clinicians and the in-charge at the primary health-care centres gave a brief talk on sickle cell disease and newborn screening to mothers presenting for immunisations. The dedicated research nurse showed parents of screened babies how to access the paediatric sickle cell programme for comprehensive care. The programme has educational materials that highlight health promotion habits and alert parents to danger signs such as fever, persistent headache, abdominal pain, vomiting and diarrhoea, the features of severe anaemia, and chest pain with breathlessness. Further genetic counselling was offered to parents who were not interested in the follow-up programme or who withdrew consent during the study.

To validate point-of-care testing in the setting of primary health-care centres, we compared test results with the HemoTypeSC point-of-care device with another frontline point-of-care screening test, SickleSCAN (BioMedomics, Morrisville, NC, USA), in a subset of children from the study population. This subgroup comprised babies who presented for routine immunisation at the five primary health-care centres within the duration of the screening period and whose mothers gave consent for additional testing. HemoTypeSC and SickleSCAN have shown high sensitivity, specificity, and accuracy as screening tests, both in laboratories and in field testing (panel).^25^, ^26^ They are both easy to use because only a heel prick is needed to obtain blood for testing. Neither point-of-care screening test needs electricity or batteries, so they can be used in primary health-care centres. Results from both screening tests can be captured in the field with a mobile phone camera for a second opinion. However, we used HemoTypeSC for screening at primary health-care centres because of the lower cost of this point-of-care screening test compared with SickleSCAN (panel). We compared results with both SickleSCAN and HemoTypeSC against the gold standard HPLC.^25^

Panel **Properties of the point-of-care tests**

**HemoTypeSC**

• Competitive ELISA
• Kit is supplied as dipsticks in a test tube, and is stable at room temperature
• Sampling technique is by finger or heel prick
• Sample reading is counterintuitive: the absence of a band is the positive result, and rigorous training is needed
• Turnaround time for result: 10 min
• Test strips can be mounted on paper with the results, but are available for comparison with the result of confirmatory testing for a limited period because the test strip paper shows sign of fragmentation with time
• Sensitivity (in field conditions): >93·8%^25^
• Specificity (in field conditions): >99·2%^25^
• Overall diagnostic accuracy: >99%,^28^ 99·1%^25^
• Cost per test: US$1·49

**SickleSCAN**

• Qualitative lateral flow immunoassay
• Kit is supplied as a cassette with tests, and can be stored for up to 2 years at room temperature
• Sampling technique is by finger or heel prick, and can be used on venous blood
• Ease of sample reading is straightforward: positive results are seen as bands on the cassette, and minimum training is needed
• Turnaround time for result: 3–5 min
• Bands do not fade on the cassette, and are available for comparison with the result of confirmatory testing
• Sensitivity (in field conditions): >94·9%^26^
• Specificity (in field conditions): >99·2%^26^
• Overall diagnostic accuracy: 99%^29^
• Cost per test: US$2·19

### Statistical analysis

We calculated the proportion of newborn babies and infants with sickle cell anaemia (HbSS), heterozygous for HbS and HbC (HbSC), with sickle cell trait (HbAS), heterozygous for HbA and HbC (HbAC), and with normal haemoglobin (HbAA). We used 95% CIs based on Fisher's exact test to reflect uncertainty related to the sample size, rather than the accuracy of the point-of-care screening test.

To check the consistency of prevalence data from our local screening programme, we compared them with data from the 2018 Nigerian Demographic and Health Survey (DHS).^30^ The Nigerian DHS is a nationally representative sample survey providing up-to-date information on a range of demographic and health indicators. Genotyping for sickle cell disease of children aged 6–59 months was done in 14 000 of 42 000 households included in the DHS. We compared our study results with those from, overall, 11 243 children and 687 infants aged 6–8 months surveyed by the DHS, using a two-proportion *Z* test, with a p value cut off of 0·05. All statistical analyses were done in R version 3.6.2.

### Role of the funding source

The funders had no role in study design, data collection, data analysis, data interpretation, or writing of the report. The corresponding author had full access to all data in the study and had final responsibility for the decision to submit for publication.

## Results

**Figure 2:**
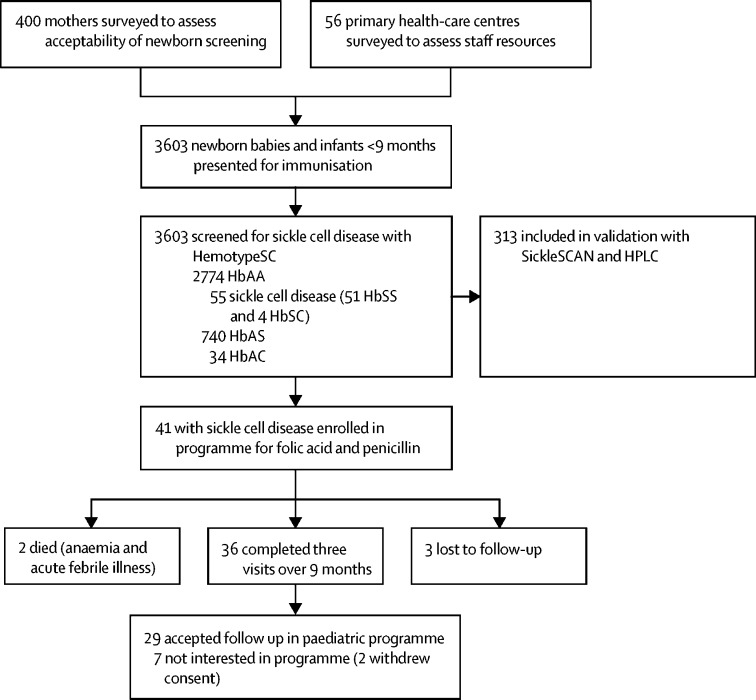
Study flowchart HPLC=high-performance liquid chromatography. HbAA=normal haemoglobin. HbAS=sickle cell trait. HbAC=heterozygous for HbA and HbC. HbSC=heterozygous for HbS and HbC. HbSS=sickle cell anaemia.

Between July 14, 2017, and Sept 3, 2019, 3603 consecutive newborn babies and infants younger than 9 months of age were screened for sickle cell disease at five primary health-care centres (figure 2). 297 (74%) of 400 mothers surveyed delivered their babies within the primary health-care centres, whereas 103 (26%) delivered outside these health facilities. 398 (>99%) of 400 mothers were in favour of newborn screening. The newborn babies screened comprised 1807 (50%) boys and 1796 (50%) girls (table 1).

**Table 1:** Characteristics of and screening results for study participants

	**Participants (n=3603)**
**Sex**	
Female	1796 (50%)
Male	1807 (50%)
**Age group**	
<6 weeks	896 (25%)
6 weeks to <3 months	1340 (37%)
3 months to <6 months	749 (21%)
6 months to <9 months	618 (17%)
**Ethnic group**	
Hausa	447 (12%)
Igbo	425 (12%)
Yoruba	336 (9%)
Ebira	228 (6%)
Igala	218 (6%)
Others	843 (23%)
Not stated	994 (28%)
Unknown	112 (3%)
Genotype*	
HbAA	2774 (77·0%, 75·6–78·3)
HbAS	740 (20·5%, 1·1–1·9)
HbAC	34 (0·9%, 19·3–21·9)
HbSC	4 (0·1%, 0·7–1·3)
HbSS	51 (1·4%, 0·0–0·3)

Data are n (%) or n (%, 95% CI). HbAA=normal haemoglobin. HbAS=sickle cell trait. HbAC=heterozygous for HbA and HbC. HbSC=heterozygous for HbS and HbC. HbSS=sickle cell anaemia.

* 95% CIs are based on Fisher's exact test and reflect uncertainty related to the sample size, rather than the accuracy of the point-of-care screening test.

The *HBB* genotype results from HemoTypeSC screening indicated 2774 (77%) children had normal haemoglobin (HbAA), 740 (21%) had sickle cell trait (HbAS), 34 (1%) were heterozygous for HbA and HbC (HbAC), 51 (1%) had sickle cell anaemia (HbSS), and four (<1%) were heterozygous for HbS and HbC (HbSC). Thus, 55 (2%) children had sickle cell disease, corresponding to one in 65 children. Of these 55 babies and infants, 41 (75%) were enrolled into a programme for free folic acid and penicillin (figure 2). 36 (88%) children completed three visits over a period of 9 months for free folic acid and oral penicillin at the pharmacy. Median follow-up for the 36 newborn babies and infants was 226 days (IQR 198–357). Two (4%) babies died; one baby died from severe anaemia when the mother was transferred to another city; the second baby developed dactylitis and an acute febrile illness and died after late presentation to hospital. Three (7%) babies were lost to follow-up because the addresses provided could not be traced.

The parents of 29 (81%) babies with sickle cell disease who were followed up for 9 months agreed to attend for further follow-up in the paediatric sickle cell programme of the teaching hospital. Seven (19%) babies' parents were not interested in further participation in the programme when contacted. Of these, two withdrew consent to participate in the study. Three babies' parents did not return after the first visit and could not be reached by telephone.

In a subset of 313 babies and infants, comparison of SickleSCAN and HemoTypeSC with HPLC showed fully consistent results across all three tests, with a specificity of 100% with HemoTypeSC, 100% with SickleSCAN, and 100% by HPLC, and a sensitivity of 100% with HemoTypeSC, 100% with SickleSCAN, and 100% by HPLC (appendix p 6). Results of screening for these 313 babies were the same for both point-of-care tests: 225 (72%) were HbAA, six (2%) were HbSS, 81 (26%) were HbAS, and one (<1%) was HbAC. None of the point-of-care screening test devices used in this study provided invalid results. The point-of-care tests were easy to use by local doctors, nurses, and community health workers.

The proportion of children with sickle cell anaemia (HbSS) in our study (1·4%, 95% CI 1·1–1·9) was consistent with data from the 2018 Nigerian DHS for children aged 6–8 months (*Z* score 0·87; p=0·17) but significantly higher than in the overall sample of children aged 6–59 months (*Z* score 2·68; p=0·0037; table 2).

**Table 2:** Comparison of screening results with those of the 2018 Nigerian DHS

	**Present study (n=3603)**	**DHS 2018, age 6–8 months (n=687)**	**DHS 2018, age 6–59 months (n=11 391)**
HbAA	2774 (77·0%)	528 (76·9%)	8782 (77·1%)
HbAS	740 (20·5%)	139 (20·2%)	2244 (19·7%)
HbAC	34 (0·9%)	10 (1·5%)	182 (1·6%)
HbSC	4 (0·1%)	1 (0·2%)	46 (0·4%)
HbSS	51 (1·4%)	7 (1·0%)	103 (0·9%)
Other	0	0	11 (0·1%)

Data are n (%). DHS=Demographic Health Survey. HbAA=normal haemoglobin. HbAS=sickle cell trait. HbAC=heterozygous for HbA and HbC. HbSC=heterozygous for HbS and HbC. HbSS=sickle cell anaemia.

## Discussion

The findings of our feasibility study show that newborn screening for sickle cell disease using point-of-care screening tests can be scaled up in local primary health-care centres by building on existing immunisation programmes, with limited additional human and financial resources for detection and follow-up in low-resource settings. Through screening of 3603 consecutive newborn babies and infants younger than 9 months, we identified 55 cases of sickle cell disease, of whom 41 were followed up for 9 months. The noted prevalence of 1·4% for HbSS and 20·5% for HbAS are somewhat lower than figures that have been used for years as representative of our populations, but they mostly accord with data in the DHS report of 2018. The higher prevalence of HbSS in our study compared with overall data from the 2018 Nigeria DHS^30^ could be a reflection of early childhood mortality because of sickle cell disease. Further work to account for differences in prevalence between ethnic groups should be done.

Newborn screening for sickle cell disease in African countries using traditional methods has so far been scant, except in Ghana.^18^, ^31^, ^32^ Screening efforts have not progressed beyond small pilot projects in local hospitals and regions, partly because of practical and financial constraints to set up and access evidence-based interventions for early diagnosis and management of sickle cell disease. Successful implementation of a newborn screening programme for sickle cell disease in African countries relies on many factors, including access to health-care facilities, easy-to-use and affordable screening devices, educational material and counselling services, and appropriate follow-up and management to prevent severe chronic complications. Our study shows the feasibility of implementing such a programme in primary health-care centres by building on the infrastructure and mobilisation associated with immunisation programmes across the African continent.

Our approach enabled us to reach out to most babies born in primary health-care centres during the study period; the reliability of the point-of-care screening test used was excellent; costs were restricted to the point-of-care screening test devices and support of children with sickle cell disease to attend follow-up appointments; and no additional staff members were needed. All these factors suggest that our approach could easily and rapidly be scaled up across Nigeria and other sub-Saharan countries. The absence of invalid point-of-care screening test results could be a reflection of the high-quality of the training provided in the primary health-care centres. Some challenges remain, such as the substantial delay in obtaining confirmatory testing from the MDG sickle cell centre in Keffi. Furthermore, some parents were reluctant (despite genetic counselling) to bring their (apparently healthy) babies for routine health maintenance visits at the teaching hospital, resulting in loss to follow-up.

The main limitations of our study are threefold. First, the number of newborn babies screened over the study period was fairly small, and just over a third were older than 3 months. No data were available for the proportion of children not attending immunisation clinics. The full schedule of vaccinations recommended by the Nigerian Federal Ministry of Health entails six visits during the first year of life, including doses delivered at birth. Newborn screening for sickle cell disease would, therefore, benefit from improvements in early access to vaccination programmes, as per these recommendations. Second, a substantial number of children with sickle cell disease could not be followed up during the study. Alongside education campaigns on the genetic nature of sickle cell disease and its clinical manifestations, it is imperative to improve access to primary health-care centres and to enhance the capacity of these facilities to provide routine health management of babies with sickle cell disease. Furthermore, many children with sickle cell disease adequately receiving immunisation and prophylactic drugs will have acute pain crises, sepsis, acute splenic sequestration, and other severe complications that cannot be managed by primary health-care centres. Referral pathways to higher levels of care for treatment of emergencies and stroke prevention with doppler scanning also need to be strengthened. Better results in follow-up could be obtained if staff at primary health-care centres are trained to do initial follow-up by a team of house-to-house mobilisers located at each facility to work within the facility catchment area. Third, evidence of the benefits of hydroxycarbamide to improve the outlook of patients with sickle cell disease, including in African settings, is now very strong. It is therefore imperative to ensure that such drugs are integrated into newborn screening programmes.

Our findings have policy implications. Our data support the integration of newborn screening into immunisation programmes in primary health-care centres, as part of universal health coverage in Nigeria. This integration would be in line with national multisectoral action plans for prevention and control of non-communicable diseases (2019–25). Furthermore, the integration of newborn screening into existing primary health-care immunisation programmes is feasible and could rapidly be implemented with limited resources in other countries in sub-Saharan Africa.
