# Correction to *Lancet Haematol* 2020; 7: e534–40

**DOI:** 10.1016/S2352-3026(20)30230-1

**Published:** 2020-07-28

*Nnodu OE, Sopekan A, Nnebe-Agumadu U, et al. Implementing newborn screening for sickle cell disease as part of immunisation programmes in Nigeria: a feasibility study.* Lancet Haematol *2020; **7:** e534–40*. In this Article, the spelling of the penultimate author's name has been corrected to “Olufunmilayo I Olopade” in the byline and relevant affiliation. The description of HemoTypeSC in the Summary and panel has been corrected to “lateral flow immunoassay”. These corrections have been made to the online version as of July 28, 2020.

