# Supplementary material for: Implementing newborn screening for sickle cell disease as part of immunisation programmes in Nigeria: a feasibility study
Source: Lancet Haematol. 2020 Jun 23;7(7):e534–40. doi: 10.1016/S2352-3026(20)30143-5 (PMC7322555; doi:10.1016/S2352-3026(20)30143-5)
Supplement: Supplementary appendix [file mmc1.pdf]

THE LANCET
Haematology

Supplementary appendix

This appendix formed part of the original submission and has been peer reviewed. We post it as supplied by the authors.

Supplement to: Nnodu OE, Sopekan A, Nnebe-Agumadu U, et al. Implementing newborn screening for sickle cell disease as part of immunisation programmes in Nigeria: a feasibility study. *Lancet Haematol* 2020; **7:** e534–40.

Supplementary Appendix

Implementing newborn screening for sickle cell disease as part of immunisation programmes in Nigeria: a feasibility study.

Supplementary Appendix.. 1

Implementing newborn screening for sickle cell disease as part of immunisation programmes in Nigeria: a feasibility study. 1

Supplementary Appendix 1: Setting up newborn screening for sickle cell disease in primary healthcare facilities 2

Supplementary Appendix 2: Consent Form for Newborn Screening 3

Supplementary Appendix 3: Protocol for using the HemoTypeSC point-of-care testing, based on manufacturer's instructions.. 4

Supplementary Figure S1: Step-by-step approach for using the HemoTypeSC test.............................. 5

Supplementary Figure S2: Interpreting the results of the HemoTypeSC test....................................... 5

Supplementary Table S1: Analysis of results of HemoTypeSC compared to HPLC................................ 6

Supplementary Appendix 4: Questionnaires used as part of this study 7

Supplementary Table S2: Vaccinations given as part of the immunization the National Programme on Immunization 8

Supplementary Appendix 1: Setting up newborn screening for sickle cell disease in primary healthcare facilities

Prior to the commencement of the screening activity, the permission and collaboration of the Health Department of the Gwagwalada Area Council, Federal Capital Territory, Nigeria were requested to inform the primary healthcare centres (PHCs) about the programme and obtain information on existing services at the PHCs including antenatal clinics (ANCs), deliveries and immunisation services.

In parallel, the opinion of key personnel and other healthcare workers from PHCs across the Gwagwalada Council Area was sought about integrating NBS with point-of-care tests into immunisation services already ongoing at the PHC. This information was used to map the PHCs in the Council Area. Participating PHCs for the NBS were selected based on numbers of patients.

For the selected PHCs, existing resources, structures and services were assessed to ascertain the feasibility of integrating NBS for SCD using a POCT as part of the immunisation programme. We used open-ended pretested questionnaires (Supplementary Appendix p 7) to collect information from the Officers-in-Charge) and other healthcare workers in the facilities and from 400 mothers using the services. One hundred per cent of the In-Charges agreed that NBS for SCD could be carried out when mothers brought their babies for immunisations in the PHCs. The above efforts paved the way for the commencement of the screening programme.

Supplementary Appendix 2: Consent Form for Newborn Screening

CENTRE OF EXCELLENCE FOR SICKLE CELL DISEASE RESEARCH AND TRAINING UNIVERSITY OF ABUJA (CESRTA)

FREE NEWBORN SCREENING FOR SICKLE CELL DISEASE (SCD)

Sample Collection site: ***Serial No: …………….**

CESRTA is organizing a **free newborn screening for sickle cell disease** in Abuja upon which certificate will be given. Please if you consent for your child to be screened and followed up, fill the following: -

Participants Name:

Date of Birth:		Age:		Sex:	

Parents Phone number:

Address:

Fathers Tribe: Mothers Tribe:

Sample collection date: …………… Sample Collected By ………………

I have read the contents of this consent form or someone has read and explained it for me and it is well understood. All my questions have been answered and I agree to have my baby's sample collected and my baby enrolled into this program for follow up if found to have sickle cell disease.

…………………………………………	…………………	…………………..
Parent's/Guardian's Name	***Signature***	***Date***
…………………………………………….	………………..	……………………
**Research Associate's Name**	**Signature**	**Date**

Supplementary Appendix 3: Protocol for using the HemoTypeSC point-of-care testing, based on manufacturer's instructions.

The following consecutive steps are illustrated in Figure S1.

1. Using dropper pipette, add six (6) drops of water to test vial. Place test vial in a compatible rack.
2. Open vial of blood sampling devices, remove one blood sampling device, and reclose vial. Obtain blood sample – a small drop is sufficient (1 to 2 microliters). Touch the white pad of the blood sampling device to blood sample, until the white pad absorbs the blood droplet. Ensure that the entire white pad has turned red.
3. Insert blood sampling device into test vial water and swirl to mix.
    - Sufficient swirling is essential for blood to be properly transferred into test vial.
    - Check visually to ensure that water has become pink or light-red in color.
    - Leave blood sampling device in the test vial after swirling.
4. Open vial of test strips, remove one test strip, and reclose vial. Insert HemoTypeSC test strip into test vial with arrows pointing down.
5. Wait 10 minutes.
6. Take HemoTypeSC test strip out of the test vial and read results. Compare test strip to the results chart (Figure S2) for reference. Red lines may appear at each of three haemoglobin variant-specific locations (HbA, HbS & HbC) and a control location. If no control line appears, the test is invalid and should be repeated. *This test is different from many other rapid tests (e.g. malaria, TB, HIV, pregnancy), because for HemoTypeSC*, the presence of a line indicates the absence of the haemoglobin and the absence of a line indicates the presence of the haemoglobin.

Supplementary Figure S1: Step-by-step approach for using the HemoTypeSC test.

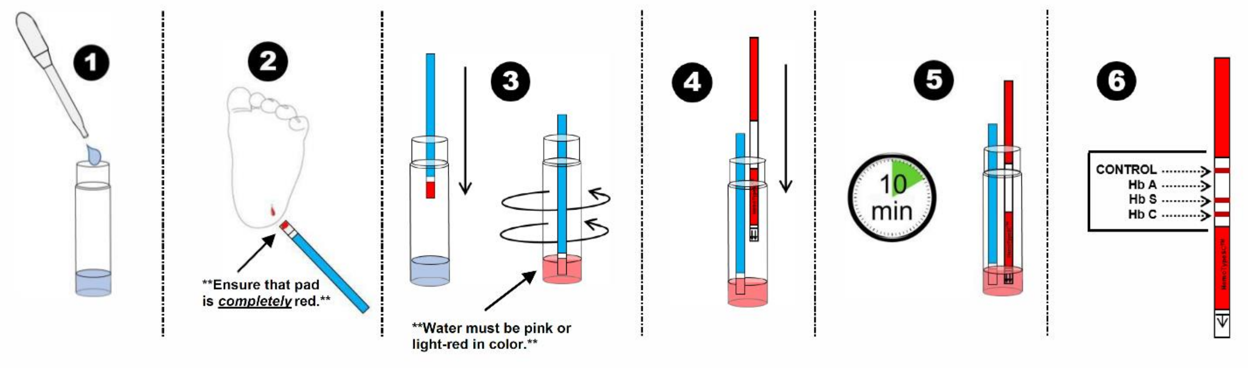

Supplementary Figure S2: Interpreting the results of the HemoTypeSC test

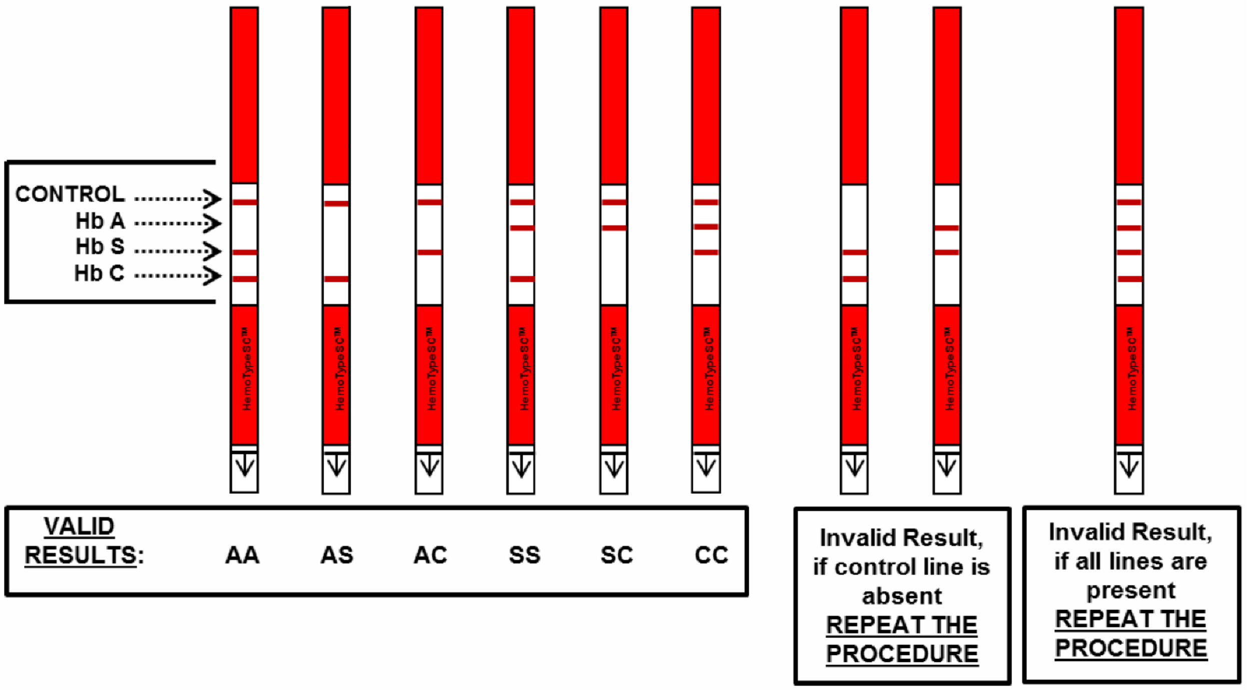

Supplementary Table S1: Analysis of results of HemoTypeSC compared to HPLC

The results of the SickleSCAN tests are not shown as they were fully consistent with those of the HemoTypeSC test.

	**TP**	**FP**	**TN**	**FN**
HbAA	225	0	88	0
HbAS	81	0	232	0
HbSS	6	0	307	0
HbAC	1	0	312	0

True Positive (TP), False Positive (FP), True Negative (TN), False Negative (FN)

Supplementary Appendix 4: Questionnaires used as part of this study

A. Questionnaire for the In-charges of the selected primary healthcare facilities

Section one: Socio-demographic information - age/date of birth, sex, marital status, tribe, cadre, year of graduation.

Section two: Total number and types of health care workers available in the PHC (midwives, nurses, community health extension workers, and laboratory personnel), knowledge of SCD, types of services available, availability of space, equipment for screening or diagnosis of SCD and knowledge of point of care screening test for SCD.

B. Questionnaire for care givers

Section one: Information on the socio-demographic data of the caregiver- Age/date of birth, sex, tribe, occupation of the mother/guardian, level of education of the mother/guardian and religion, number of children of the mother/guardian, home address (exact location with description of important landmarks, contact telephone, type of phone)

Section two: Information on the knowledge of the mother/guardian about SCD, haemoglobin genotype status, knowledge of newborn screening and acceptability of newborn screening for early diagnosis and intervention for SCD in newborns.

Section three: Information on the outcomes of the point of care screening test (haemoglobin genotype AA, AS, AC, SS, SC and indeterminate)

Supplementary Table S2: Vaccinations given as part of the immunization the National Programme on Immunization

AGE	DISEASE	VACCINE/ANTIGEN	SITE
AT BIRTH	Hepatis B<br>Polio<br>Tuberculosis	HBV 1,<br>CPV0<br>BCG	Upper part of the thigh<br>Oral<br>Upper left arm
6 weeks	Polio<br>DPT, HIB, Hepatitis B<br>Rotavirus<br>Pneumonia & Otis Media	CPV 1<br>Pentavalent 1<br>Rotarix 1<br>Synoflorix 1 or<br>Prevnar 13	Oral<br>Upper part of the thigh (right)<br>Oral<br>Upper part of the thigh (left)
10 weeks	Polio<br>CPT, HIB, Hepatitis B<br>Rotavirus<br>Pneumonia & Otis Media	CPV 2<br>Pentavalent 2<br>Rotarix 2<br>Synoflorix 2 or<br>Prevnar 13	Oral<br>Upper part of the thigh (right)<br>Oral<br>Upper part of the thigh (left)
14 weeks	Polio<br>CPT, HIB, Hepatitis B<br>Pneumonia & Otis Media	CPV 3<br>Pentavalent 3<br>Synoflorix 1 or<br>Prevnar 13	Oral<br>Upper part of the thigh (right)<br>Upper part of the thigh (left)
6 months	Vitamin A	Vitamin A	Oral
9 months	Measles, Yellow Fever	Measles,<br>Yellow Fever	Upper part of the thigh (right)<br>Upper part of the thigh (left)
